# MicroRNA Modulation during Orthodontic Tooth Movement: A Promising Strategy for Novel Diagnostic and Personalized Therapeutic Interventions

**DOI:** 10.3390/ijms232415501

**Published:** 2022-12-07

**Authors:** Giovanni Cultrera, Antonino Lo Giudice, Simona Santonocito, Vincenzo Ronsivalle, Cristina Conforte, Giuseppe Reitano, Rosalia Leonardi, Gaetano Isola

**Affiliations:** Department of General Surgery and Surgical-Medical Specialties, School of Dentistry, University of Catania, Via S. Sofia 78, 95124 Catania, Italy

**Keywords:** miRNAs, osteogenic differentiation, molecular medicine, interventions, orthodontic tooth movement, cellular pathways, biomolecular medicine

## Abstract

The Orthodontic Tooth Movement (OTM) is allowed through a mediated cell/tissue mechanism performed by applying a force or a pair of forces on the dental elements, and the tooth movement is a fundamental requirement during any orthodontic treatment. In this regard, it has been widely shown that each orthodontic treatment has a minimum duration required concerning numerous factors (age, patient compliance, type of technique used, etc.). In this regard, the aim of the following revision of the literature is to give readers a global vision of principal microRNAs (miRNAs) that are most frequently associated with OTM and their possible roles. Previously published studies of the last 15 years have been considered in the PubMed search using “OTM” and “miRNA” keywords for the present review article. In vitro and in vivo studies and clinical trials were mainly explored. Correlation between OTM and modulation of several miRNAs acting through post-transcriptional regulation on target genes was observed in the majority of previous studied. The expression analysis of miRNAs in biological samples, such as gingival crevicular fluid (GCF), can be considered a useful tool for novel diagnostic and/or prognostic approaches and for new personalized orthodontic treatments able to achieve a better clinical response rate. Although only a few studies have been published, the data obtained until now encourage further investigation of the role of miRNA modulation during orthodontic treatment. The aim of this study is to update the insights into the role and impact of principal micro-RNAs (miRNAs) that are most frequently associated during OTM.

## 1. Introduction

Orthodontic tooth movement (OTM) is a process in which mechanical force induces alveolar bone resorption, mediated by osteoclast, on the pressure side and alveolar bone deposition on the tension side [1,2]. The duration of orthodontic treatment may vary based on several factors, including the modulation of microRNA (miRNA) expression, that, in this context, may occupy an important role. miRNAs are small noncoding RNA regulating gene function at the post-transcriptional level by pairing with 3′UTR of target mRNAs [3]. The identification of miRNAs commonly associated at OTM process, might have a significant impact both for personalized treatment and/or prognostic strategies.

Today, orthodontic treatment is becoming more popular; however, the biological mechanisms underlying OTM are still not completely understood. Basically, OTM is allowed by a gradual application of mechanical force that leads to a complex combined resorption/deposition mechanism of bone tissue at the periodontal tissue level [1]. The tooth to alveolar bone relationship is mediated by the periodontal ligament (PDL), a support structure for the teeth made up of many biological components and principally cells, collagen fibers, vessels and nerves immersed in a fibrous connective tissue [4]. The tissue reactions shown during physiological tooth movement and those during orthodontic tooth movement are basically equivalent. The tissue changes caused by orthodontic forces are more evident and widespread because the teeth are moved more quickly during OTM. The development of a simultaneous bone resorption mechanism that allows the dental element to move a certain direction is a fundamental prerequisite for orthodontic movement. The relationship between these biological micromolecules (miRNAs) and the mechanism underlying the OTM looks obvious because miRNAs give us evidence of biological activities of cells contained in biological tissues surrounding teeth.

MicroRNAs, that are composed by approximately 20–25 nucleotides in length of non-coding RNA molecules, are considered as pivotal regulators of gene expression during various physiological and pathological cellular operations [5]. Moreover, they are secreted and can be isolated in numerous biological fluids (i.e., CGF) and has been revealed to be concentrated in exosomes [6,7]. According to several authors, it may be important to evaluate their biological presence because various studies have shown their role during osteoclastogenesis process [8,9]. The case of Sugatani et al. demonstrated how OC differentiation decreased from OC precursors without miRNAs activity [10]. It is well known that miRNAs perform a key role in remodeling bone process by controlling the osteoclast and osteoblast differentiation [11]. Several miRNAs have been studied in patients, in vivo and in vitro to evaluate their principal role following the application of orthodontic forces or systems capable to simulate OTM to assay their effects on the target genes and pro-inflammatory mediators (receptor activator of nuclear kappa ligand RANKL, osteoprotegerin OPG, cytokines, etc…) that allow the differentiation of osteoclast/osteoblast cells during the bone remodeling [12,13]. Considering the quantity of miRNAs already present in the crevicular fluid of patients undergoing a particular orthodontic therapy, it is possible in dentistry field to use these molecules also for other diagnostic and therapeutic purposes.

It is well known that the fundamental requirement for having an orthodontic tooth movement is to obtain a partial bone remodeling of the alveolar bone tissue in contact with the root of the tooth subjected to force. The early stages of OTM are due to acute inflammation, i.e., vasodilation and increased vascular permeability to lymphocytes [14]. At a time later, bone remodeling is regulated through a series of complex processes carried out by many biological mediators that allow a cross-talk between bone tissue cells (osteoblasts and osteoclasts), with the ultimate goal of having a new balance between bone apposition and bone removal [15]. The modulation of bone resorption is influenced by RANKL/OPG ratio. Receptor activator of nuclear factor kappa B ligand (RANKL) promotes osteoclast differentiation due to the link with RANK (receptor activator of nuclear factor kappa B); while osteoprotegerin (OPG) is a natural inhibitor of osteoclasts as it reduces the association between RANKL/RANK (antagonist role), thus suppressing the bone resorption function [16,17,18]. As a matter of fact, in an in vitro study conducted by Nishijima et al. on PDL cells the RANKL/OPG ratio increased in the compression zone during OTM, while it was decreased in the tension zone due to the increased secretion of OPG [19,20]. To underline that miRNAs are involved in the regulation of the inflammatory mechanism underlying bone remodeling, some studies show that miRs-21-145-146a-150 and -200 were also reported to be involved in the regulation of OPG balance and in the recruitment of osteoclasts, favoring the polarization and activation of these cells [21,22].

miRNAs have been thoroughly investigated in oncology and relevant information has come to light regarding their potential use as biological markers for a variety type of cancer. Furthermore, it has been established that miRNAs can be used as biomarkers in oral cancer because they are associated with the possibility neoplastic transformation of oral premalignant lesions to malignancy [23]. In dentistry, some miRNAs are used as prognostic and diagnostic markers in numerous pathologies (OSCC oral squamous carcinoma, periodontal disease, homeostasis and craniofacial malformations, such as CLP, etc…) [24,25,26]. miRNAs have also been noticeable in other dental diseases, such as pulpitis and periodontitis [27,28]. According to research conducted by the group of Scribante et al., the long-term clinical conditions of patients can be improved by the addition of adjuvant therapies (ozone therapy and photobiomodulation PBM) to routine mechanical debridement (SRP) [29]. In this regard, it would also be fascinating to investigate the modulation of miRNAs after the combined treatment of SRP plus ozone therapy and photobiomodulation (PBM) in order to obtain additional indicators on the state of periodontal health in terms of the intensity of inflammation, particularly for those patients who are selected to receive orthodontic treatment in addition to periodontal therapy. The study of miRNAs biology has greatly evolved in the nearly two decades since the first miRNA was discovered. The findings from the present review indicate that their application scope is enlarged to include both oncology and dentistry. miRNAs have become important tools and targets for new therapeutic methods as a result of studies regarding the roles they play in development and disease, particularly cancer. They may also be crucial tools in the execution of novel or more efficient orthodontic treatment. Additionally, in the field of orthodontics, the study of miRNAs has assumed great importance in the last decade. Orthodontics, fixed and mobile, has improved its techniques in recent years, allowing for the achievement of performing more objectives than in the past. In this regard, molecular biology also makes its contribution to this field of dentistry, thanks to the study of the modulation of miRNAs during OTM. In particular, what appears to be their function during certain tooth movements [30,31,32,33,34] or their role in the modulation of biological mechanisms [35,36,37,38,39,40,41,42,43,44] can provide a fundamental tool to the future orthodontists for a new approach to the techniques that exist in this field of dentistry. However, the expression of miRNAs during orthodontic movements remains to be further investigated.

The aim of the study is update the insights of the role and impact of principal micro-RNAs (miRNAs) that are most frequently associated during OTM. Anyway, the evidence for post-transcriptional regulation of the accompanying alterations by the miRNAs is still scant, despite the fact that numerous biomarkers have been discovered in oral fluids at the biochemical level. In order to understand the biology of hard and soft tissue modifications and remodeling, it is necessary to discover the most relevant miRNA biomarkers in OTM, their modification during the time and their influence on the gene expression profile and target proteins.

## 2. Materials and Methods

The PubMed search using “Orthodontic Tooth Movements” and “microRNAs” key words was performed to find previous studies describing tooth movements and the role of microRNAs. Twelve relevant studies were selected: three in vitro studies, three in vivo studies, three studies both in vivo and in vitro, one clinical trials and in vitro studies and only two studies exclusively clinical trials.

## 3. Results

### 3.1. Relationship between miRNA and OTM

#### 3.1.1. Modulation of miRNAs in Canine Retraction

In a recent 2021 review of the literature [30], Kapoor et al. highlighted miRNAs correlated with a specific teeth movement: canine retraction (through CGF samples).

In the first study, an increase in miRNA-29 (a/b/c) levels was observed in the interval T0 (before retraction)–T4 (after 6 weeks), evidence that this family of miRNAs can play a fundamental role in regulation of osteoclasts and molecules of the extracellular matrix [31].

The second study demonstrates a decrease in miRna-34a and an increase in MMPs-2, 9 and 14 in the period T2 (1 day after retraction)–T4 (4 weeks after retraction) and this displays a correlation between this type of miRNA and OTM, osteogenic regulation and the Wnt/b-catenin pathway [32].

In the third study, the family of miRNAs 29 (a/b/c) undergoes an increase in the intervals T1 (before retraction)–T5 (5 weeks later), except for miRNA-35c which has only a peak of decrease in T3 (1 day after retraction). Other miRNAs studied (21, 101) also showed the same statistically significant increase. The author concludes by considering the miRNA-29 (a/b/c) family and miRNA 21 involved in the regulation processes of osteoclasts and osteoblast; instead, the miRNA 101 against fibrogenesis [33].

The fourth and latest study underlined the role of miRNA-27a/b, 146a/b and 214 in OTM. A positive correlation was demonstrated between the distance of canine retraction in the T1 interval (before coil spring activation); T2 (2 weeks after retraction) in the expression of miRNA 27a/b and 214 at T2; in the T1–T3 interval (5 weeks) of miRNA 27b at T3, no change in miRNA 146a/b, mild negative correlation between CGF volume samples; and miRNA 27a changes at T4 (7 weeks) [34].

#### 3.1.2. The Role of miRNA-21 during OTM

- Yuanyuan Zhang et al. investigated the role of miRNA-21 on four groups of rats: TM (Tooth Movement), PAOO (Periodontal Acceleration Orthodontic Osteogenesis), AgomiR-21 (MiR-21 Overexpression) and AntagomiR-21 (inhibition of miR-21). After 7 days of treatment, the tooth displacement obtained by the agomir-21 group was significantly greater than both TM and PAOO, demonstrating the role of miRNA-21 in promoting RANKL-mediated osteoblast differentiation via negative modulation of the PDCD4 gene and thus facilitating OTM [35].
- This study provides a demonstration of the miRna-21 increase in periodontal ligament cells (PDL) and an acceleration of orthodontic movement in mice treated with *E. Coli* LPS inoculation. This suggests a direct role of miRNA-21 during OTM even in an inflammatory microenvironment [36].
- It has also been shown that the role of miRna-21 on OC differentiation is not only due to the modulation of the PDCD4/C-fos pathway, but also due to the influence of RANKL secretion by T cells [37].

#### 3.1.3. miRNA-34a

- An in vitro study of periodontal ligament stem cells (PDLSC) obtained from the periodontal ligament (PDL) of teeth extracted for orthodontic reasons demonstrated the inhibitory role of miRNA-34a and miRNA-146a against osteoblast by silencing the CELF3 gene [38].
- In contrast, Wenwen Yu et al. showed a positive role of miRNA 34a in osteogenic differentiation both in vitro and in vivo. The miRNA-34a, provided by the *N*-Ac-l-Leu-PEI vector, is capable of increasing dental anchorage in vivo and stimulating osteogenic differentiation in vitro. Its target gene is Gsk-3β which promotes the phosphorylation of the β-catenin protein, which is essential once accumulated in the cell nucleus to favor the Wnt/beta-catenin pathway and, therefore, the activation of OB differentiation gene [39].

#### 3.1.4. Other miRNAs Implicated on OTM

- Other authors performed studies about the evaluation of miRNAs associated with frequent complications of orthodontic treatment, i.e., root resorption. miRNA-155-5p significantly decreases with increasing degree of root resorption (a sign of excessive osteoclast activation), so this miRNA could be implicated in the inhibition of osteoclast differentiation by suppressing transcription and expression of CXCR2 gene, involved in the synthesis of many OC enzymes linked to bone resorption [40].
- Wendan He et al. demonstrated that miRNA 125a-5p promotes M2 polarization of macrophages, which increases osteogenesis. Furthermore, this miRNA strongly increased under an orthodontic force and the OTM role is performed through the inhibition of the ETV6 gene [41].
- Considering that rBMSCs subjected to mechanical stress undergo osteogenic differentiation, the in vitro decrease in miRNA-503-5p levels (minimum level after 12 h) indicates its negative role in this type of differentiation. Furthermore, the confirmation occurred in vivo as the levels of this miRNA significantly decreased in the tension side (trend maintained for 3 days) and then increase again [42].
- A further study related the expression of miRNAs in the two sites identified during an orthodontic movement, namely compression and tension side. The authors demonstrated that miRNA-3198 is overexpressed on the compression side and at the same time there is a reduction in OPG levels in hPDL cells (as opposed to on the tension side). These results suggest that miRNA-3198 is capable to inhibit OPG (target) in response to a mechanical stimulus [43].

The last study performed using high-throughput sequencing shows an analysis of 47 known miRNAs: 31 were upregulated (involved in mechanical force-induced osteoblastic/cementoblastic differentiation in PDLCs) and 16 were down-regulated in the stretched PDLCs compared with control. Furthermore, the target genes upregulated in PDLCs after force stimulation are RUNX2 (from miR-218-50, miR-625-3p, miR-150-5p, miR-2682-5p, miR-7-1-3p, miR-221-3p and miR-133a-5p), OSX (from miR486-3p, miR-145-3p, miR143-5p and miR-133a-3p), DLX5 (from miR-376a-3p and miR-942-3p), MSX2 (from miR-7-1-3p, miR-708-5p and miR-942-3p) and SATB2 (from miR-218-5p, miR-1-3p, miR-31-5p and miR-376a-3p); only RUNX2, MSX2 and SATB2 were upregulated also in tension force loading PDLCs [44]. In another in vitro study where PDLCS cells were subjected to cyclic tension for 72 h, miR-195-5p was also found to be downregulated and negatively correlated with osteogenic differentiation [45]. The findings make it clear that there are not enough studies on miRNAs linked to OTM. What is apparent is that there is a wide range of differences in how they are applied in the orthodontic field, which prevents the creation of standards for their application in clinics. We have chosen to schematize them in a table with the following connotations: name of the miRNA, role in OTM, type of study and biological sample analyzed in order to help readers comprehend their functions and have it possible that their functions have been emphasized thus far.

Below are reported the most relevant studies describing the role of miRNAs in OTM (Table 1).

## 4. Discussion

In order to accurately identify their targets, a critical assessment of the evidence relating to the existence and alteration of miRNA levels in OTM studies was performed in the present review. According to what emerged from the various studies, several miRNAs are expressed differently during OTM. Unfortunately, the role performed by miRNAs in bone resorption to allow tooth movement has not been fully understood [46].

As we know from previous studies, the miRNA-29 family promotes osteoclastogenesis involving TNF-alfa [47,48]. In fact, during OTM, TNF-alpha of human periodontal ligament fibroblasts are also increased on the compression side and this may contribute to stimulate osteoclast differentiation by influencing RANKL levels [49]. Regarding the type of orthodontic movement studied, only four papers were performed taking into consideration the canine retraction and the miRNAs related to this type of tooth movement are the family of miRNA 29 (a/b/c) and other miRNAs (21, 27a/b, 34a, 34c, 101, 146a/b and 214). The miRNA-29 family regulates the bone remodeling process through the modulation of osteoclasts, osteoblasts and some extracellular matrix molecules [50,51,52]. Franceschelli et al. demonstrated that miRNA-29 regulates osteoclast survival by modulating both targets, such as the calcitonin receptor (Calcr), nuclear factor I/A for macrophage differentiation (Nfia) and G protein coupled to the receptor 85 (Gpr85), but also other targets for the cytoskeleton modulation [51,52,53]. However, other authors have demonstrated the opposite role of miRNA-29 in modulating osteoclastogenesis by inhibiting RANKL and by stimulating osteoblast differentiation [54,55]. Our review showed that miRNA studies performed in relation to OTM were conducted exclusively during canine retraction and among all those examined only miRNA-29 family appears to perform a predominant role. Although, a study by Li et al. could support the role of miRNA-101 in canine retraction by targeting TGF-beta and PLAP-1 (periodontal ligament-associated protein-1), which are implicated in the process of bone deposition [56]. Regarding miRNA-146, it is also involved in the canine retraction of the present review and its return to initial values could be due to its negative control role against inflammation through the suppression of the MAPK (pro-inflammatory mitogen-activated protein kinase) and the NF-kB pathway [57]. In terms of canine retraction procedures, principally miRNA 29, but also miRNAs 101 and 104, seem to be most involved in this movement. Unfortunately, their precise function has not yet been fully understood, thus more research is required.

The main part of studies performed on OTM/miRNAs underlined the priority role of miRNA 21 and 34a.

**Figure 1:**
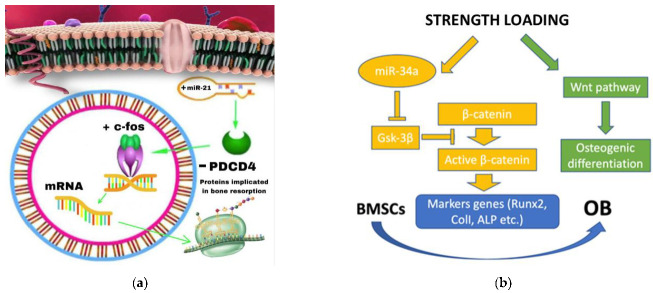
(**a**). Through the suppression of its target PDCD4 (Programmed Cell Death 4), which is caused by an intracellular increase in miRNA-21, the transcription factor c-fos is stimulated, thus increasing the protein synthesis involved in bone remodeling. (**b**). Summary scheme of one of the action mechanisms of miRNA-34a by which, following the application of a loading force in vitro and in vivo, allows osteogenic differentiation and OB (Osteoblast) differentiation from BMSCs (Bone Marrow Stem Cells). The arrows indicate a stimulating effect, the blunt ones instead of inhibition.

Previous in vitro studies have revealed that a high amount of miRNA in osteoclast precursors promotes their RANKL-mediated differentiation [10,11,12,13,14,15,16,17,18,19,20,21,22,23,24,25,26,27,28,29,30,31,32,33,34,35,36,37,38,39,40,41,42,43,44,45,46,47,48,49,50,51,52,53,54,55,56,57,58]. It has already been shown that miRNA-21 responds to inflammatory signals in bone marrow stem cells [59] and, therefore, is one of the most implicated miRNAs during OTM. miRNA 21 plays a role in both direct and indirect tooth movement. In fact, it is able (in a direct way) to control osteoclastogenesis in osteoclasts [36]. The PDCD4 gene is the most accredited target of miRNA21 [60]. This predominant role in bone homeostasis mechanisms is supported by Hong Wang et al. who demonstrated how RNA-21 contributes to bone reconstruction in jawbone defects and a deficiency of miRNA-21 can impair bone regeneration [60]. Indirectly, indeed, miRNA 21 is capable to regulate, through the mechanism of bone remodeling mediated by OC differentiation, the release of greater quantities of RANKL by T cells of the immune system [36]. Furthermore, T cells control OTM via Th1-related cytokines, whose regulation is promoted by miRNA-21 at the expense of Th2 phenotype [61,62,63,64]. miRNA-21 is also implicated in some surgical procedures (Periodontally Accelerated Osteogenic Orthodontics, PAOO), which significantly reduce treatment times. PAOO is also associated with numerous clinical advantages including a reduction in root resorption, as the miRNA that inhibits the differentiation of osteoclasts by targeting CXCR2 is miRNA-155-5p [65,66]. However, no studies have been conducted considering the role of miRNA-155-5p in PAOO. In addition to dentin phosphoproteins (DDP), miRNA-155-5p can also be considered a valid marker of root resorption as its concentrations in CGF samples are consistent but opposite to those of DDP [42]. Instead, CXCR2 is a G-protein-coupled receptor found on the surface of many cells, including macrophages, osteoclasts and osteoblasts, and can bind IL-8 and performs an important role in OC differentiation mediated by RANKL [40,67,68]. IL-8, like other pro-inflammatory cytokines, has a pivotal role in the inflammation process and during the OTM changes of IL-8 in CGF samples reflect the state of bone resorption and periodontal inflammation. [69,70]. These results suggest the effective role of miRNAs not only as active at the post-transcriptional level of mRNAs and therefore proteins, but also involved by targeting other biological mediators (cytokines). Curtale et al. have shown that miRNAs are closely related to macrophage polarization [71,72], indeed, miRNA-125a-5p exerts its role by promoting the macrophages polarization towards an M2 phenotype by targeting ETV6 [40]. Basically, the miRNA-21 action mechanism consists in the negative interaction with its target gene PDCD4, which directly inhibits the activity of the transcription factor protein-1 AP1 (decisive in the synthesis of miRNA-21) [73]. This leads to an increase in the transcription factor C-fos levels, which is essential for promoting osteoclastogenesis [74,75]. Furthermore, previous works have revealed the foremost role of miRNA-21 in OCs differentiation, as a matter-of-fact, miRNA-21 silencing inhibits bone resorption and an in vitro study show that cells treated with TNF-alpha/RANKL, which are associated with an increase in osteoclastogenesis, also manifest a greater expression of miRNA-21 [10,47]. Hence, miRNA-21 overexpression is a determining factor to increase orthodontic movements. These studies reveal the primary function of miRNA-21 in the control of bone homeostasis, which ensures a correct balance between bone resorption and bone deposition, especially after an orthodontic movement. However, miRNAs in general have been studied in oncology because they are often overexpressed in some cancers, as in the case of miRNA-21, and its predominant role in promoting cell invasiveness in renal cell carcinoma through the PDCD4/c-jun pathway (AP-1) [76,77] (Figure 1a). From this, it is clear that miRNA-21 occupies a leading role in the control of bone homeostasis through numerous mechanisms during bone remodeling and therefore during OTM; however, the other mechanisms of action are not still clear and they need to be investigated further.

It has been amply demonstrated that the expression of some miRNAs is correlated in various stages of odontogenesis [78]. These studies include miRNA-34 which is also capable of inhibiting bone metastases and osteoporosis by suppressing osteoclastogenesis [79]. Given that metalloproteinases occupy a fundamental role in numerous physiological processes (inflammation, repair and development), including bone remodeling/resorption, during OTM, miRNA-34a promotes orthodontic movement by suppressing MMP-s 2, 9 and 1; moreover, the Wnt/b-catenin pathway is involved in this process [32,80]. In fact, confirming the negative modulation of MMPs after the application of an orthodontic force, show that the levels of miRNA-34a are profoundly reduced both in the tension side and in the pressure side and this is in agreement with the role performed by the activated osteoclasts which, once they reach the matrix bone surface, increases the release of proteolytic enzymes, including MMP-9, to digest the organic matrix [32,81]. In a recent study, authors found that MMP-9 is expressed on OCs absorbed from the root of bovine deciduous teeth [82]. In a 2022 paper, performed exclusively in vitro on PDLSC cells subjected to cyclic stress, miRNA-34a and miRNA-146a were under-expressed and thus unable to downregulate their target gene CELF3 [38]. The application of a strength loading, in vivo and in vitro, on the one hand promotes the WNT/beta pathway, and therefore osteogenic differentiation, and on the other hand it causes an increase in miRNA34a levels which, through the inhibition of its target protein GSK-3beta, promotes the activation of beta-catenin that promotes the transcription of its marker genes (Runx2, Coll, ALP, etc.) which ultimately favor the differentiation of osteoblasts from BMSCs (Bone Marrow Stem Cells) [39] (Figure 1b). Instead, miRNA-103a works in the opposite direction to miRNA-34a because it inhibits bone formation by targeting RUNX2 [83]. In the same study, miRNA-34c was also over-expressed, while miRNA-29b, miRNA-30a and miRNA-188 were under-expressed in stretched BMSCs. Other miRNAs have also been identified as modulators of the transcription factor RANX2. Furthermore, an in vivo and in vitro overexpression of miRNA-503-5p in BMSCs attenuates osteogenic differentiation and decreases the expression of both RANX2 and ALP; miRNA-103a is downregulated during osteoblast differentiation by cyclic mechanical stress-induced with consequent increase in RANX2 by targeting pank3 gene [45,84,85,86,87,88]. What emerged from the research results is that miRNA 34a appears to have a contrasting role since on the one hand it inhibits osteoblasts by silencing the CELF3 gene but on the other it stimulates osteogenic differentiation both in vivo and in vitro. It has already been ascertained that miRNAs 34a has a fundamental role among all the miRNAs studied, however there are no studies in the literature that show whether the main role of this miRNA is positive or negative towards osteogenic differentiation and therefore in favoring or not the OTM. These doubts arise spontaneously the need to undertake new studies in the future to clarify to the scientific community the primary role of this miRNA or whether it may be dependent on other specific local conditions. Ultimately, the miRNA-34 family, together with the other miRNAs listed, seem to be the most involved together with miRNA-21 in the modulation of bone resorption also through the involvement of MMPs. However, due to the lack of studies in this area, more research is necessary to determine the role of the miRNA-34 family and the other miRNAs in question.

In previous investigations, researchers evaluated the biological role of miRNAs as mechanosensitive agents. Indeed, Marin et al. focused on the mechanosensitive role of miRNAs in vascular endothelial cells during shear stress-regulated endothelial responses [84]. This might be connected to the fact that during OTM a vascular compression is applied to the vessels of periodontium, which could have impact on the expression of some miRNAs that modulate tissue proliferation. In addition, in OTM tension sides, miR-503-5p was identified to function with a mechano-responsive role which can inhibit osteogenic differentiation of bone marrow-derived mesenchymal stem cells (BMSCs) under mechanical stretch and bone generation [85].

As it is known, during an orthodontic movement, two zones are created in the alveolar bone called the tension side and the compression side, respectively. In the compression side the bone must be removed to allow space for tooth movement, in the tension side the bone must be removed to fill the gap left by the tooth [86]. Based on this premise, we can consider the two zones differently and we can expect a different modulation of miRNAs in the compression and tension side. To confirm this premise, Kanzaki et al. demonstrated that miRNA-3198 is up-regulated in the compression side and down-regulated in the tension side [43]. Furthermore, in human lung tissue cancer, miRNA-3198 has also been recognized [87]. The lack of modulation difference between the two sides may be because in some tooth movements the two tension and compression sides exist simultaneously on the two sides of tooth movement. Only in some movements (body translation) this does not occur, because we have the tension side on the one hand and the compression side on the other. We can believe that if this distinction regarding the different modulation of miRNAs between the two displacement sides is not evident, it could be due to the aforementioned coexistence of two zones in the same side. This would simply cancel the hyperregulation or downregulation of the miRNA under consideration or make one or the other prevail expression in relation to the physical extension of the two zones. On the other hand, the lack of separation of the two displacement sides, corresponding to the compression and tension side, is also because the few studies carried out do not make a distinction between the various types of dental movement taking into consideration only the canine retraction in humans or the mesialization of the first maxillary molar in mice. It is interesting to note how Xinqi Huang et al., in a recent 2020 review, analyzed the action of miRNAs in bone remodeling through their delivery in extracellular vesicles (EVs) [88]. Membrane proteins, double strand of lipid and intravesicular payloads are components of EVs that are released by mother cell at one location, diffused into the bloodstream and then bind to target cells at another location (either close by or far away) to release their contents [89,90]. Of all the possible contents of these EVs, miRNAs catch our attention most of all. Since EVs can mirror the phenotypic of the cell from which they originate, when stem cells (MSCs) produce them, they can transmit genetic information to control the development of other stem cells (precursors of osteoblasts) [91]. From this, it would explain the possibility of obtaining a greater migration of osteoblasts in subjects affected by pseudarthrosis in case of transplantation of EVs derived from BMSC [92]. The fact that MSCs are a heterogeneous cell type means that EVs generated from MSCs might also be heterogeneous, have varying miRNA concentrations and have varying impacts on their targets [93]. The information exchange between osteoblasts and osteoclasts is known to be crucial for preserving the homeostasis of bone tissue. Through direct cell-to-cell contact, extracellular ECM interaction and cytokines, osteoblasts may influence in one direction osteoclast development, differentiation, and apoptosis and the same thing may take place in the opposite direction [94]. Recent research has revealed that EVs-miRNAs produced by osteoblasts may fuse with osteoclasts to stimulate osteoclastogenesis and accelerate the removal of damaged tissue during bone healing; furthermore, it was also demonstrated the ability of RANK-rich EVs produced from osteoclasts to prevent osteoclastogenesis by suppressing the RANK-RANKL interaction in osteoblasts [95]. One of the functions of miRNAs is to interfere with MMPs. Similar to how rheumatoid arthritis damages joints, miRNA-23a inhibits MMP-3, which results in the deterioration of several ECM components and, by inhibiting the production of RUNX2 and promoting the expression of lncRNA MT1DP that is mediated by YAP1, the same miRNA found in EVs could successfully decrease osteogenesis [96,97]. It is interesting to note the potential relationship between muscle activity and bone modeling, possibly mediated by miRNAs. It is suggested that EVs produced from some myoblasts could encourage the differentiation of pre-osteoblastic cells into mature osteoblasts by activating of β-catenin pathway and that could lead to an increase in osteogenesis [98]. Due to the great biocompatibility of EVs, and to lack of the major histocompatibility complex proteins on the cell surface (MHC-I and MHC-II), that it has been discovered that the miRNAs they transport may serve as particular biomarkers for various bone disorders [99,100]. These investigations allow us to make the hypothesis that EVs-miRNAs could be utilized in genetic engineering approaches to execute a gene regulation that can enhance medical treatments that influence bone tissues, such as in the case of orthodontics. The subject is still not fully developed, thus further research is required to improve the application of these strategies. In support of our study on miRNAs in the orthodontic field, it is also worth mentioning other studies carried out on the possible pathogenetic role of these non-coding mRNAs in some congenital developmental anomalies. This is the example of the relationship between miRNAs and secondary palate development that led to the identification of miRNAs dysregulation in cleft lip and palate (CLP) cases, specifically 57% were upregulated and 47% downregulated and furthermore, patients with cleft palate (CP) showed altered expression of 9 miRNAs [101,102,103]. With the development of bioinformatics, the gap between basic research and applied disciplines in medicine or dentistry has demonstrated to be extremely small. In a recent systematic review from 2018, STRING bioinformatics analyzes were employed to investigate the most important signaling pathways and protein-protein interaction (PPI) networks and thus delineate adaptation to cellular stress in vitro [104]. According to bioinformatic analyses of target genes in response to mechanical stresses in vitro, hypoxia may also be a distinguishing factor in osteocyte differential response [105]. Furthermore, Iwasaki et al. imagined continued research fusing the discipline of orthodontics with genetics and biology to improve the achievable objectives of orthodontic treatments. Thanks to bioinformatics, clusters of DEG (Differential gene expression) genes were discovered through an in vitro study done on rats. One cluster shows a downregulation on the third day, then an increase up to the fourteenth day (safety linked to cell proliferative events), while the other presents an initial peak and a decrease up to the fourteenth day (definitely linked to the innate/adaptive immune-mediated response, etc.) [106]. Future study on OTM biology and remodeling can build on this understanding, supporting the ideas of precise therapy for tooth acceleration and movement without iatrogenic adverse effects. 

We note at the conclusion of our discussion that one clinical application of miRNAs might be to evaluate their concentration in crevicular fluid in order to quickly comprehend what appears to be the efficacy of the bone remodeling mechanisms and, as a result, to be able to more accurately modulate the dosage of orthodontic forces. However, from our research, miRNAs could have a higher clinical impact than tooth movements if employed in patient evaluations for orthopedic movements (such as Rapid Maxillary Expansion RME), particularly if borderline and consequently toward the end of growth. miRNAs analysis may be a useful tool when it comes to knowing the metabolic activity of the bone and determining when to apply and how long to maintain orthopedic forces on patients. These factors can be quite helpful, but we have to recommend performing additional research in this area in order to improve knowledge and give clinicians more specific instructions.

In conclusion, another pivotal consideration for us in our review is that the research of probiotics in medicine, which has generally become very important in recent years. Many academics have concentrated their attention on the topic, as in the case of the Butera et al. group who evaluated the effect of probiotics in the periodontal patient and during periodontal therapy [107,108]. Given that the microenvironment surrounding a dental element undoubtedly affects the OTM and that numerous studies in the literature support the use of probiotics to balance any dysbiosis present in the oral cavity, it may be of interest to further explore how these treatments may affect the movement of the teeth and thereby serve as an additional tool to better treat both periodontal and non-periodontal patients.

The possibility of a novel interpretation of the function of miRNAs in orthodontics is emphasized by this observation, which will make it possible to conduct future research with an increased awareness of the clinical case and to reduce the risk of mistakes in the evaluation of miRNAs during OTM. Despite studies performed in vivo, in vitro and through clinical trials, the mechanisms underlying the interaction between miRNAs and orthodontic movement are still not well understood by the scientific community. For this reason, we suggest to conduct further studies in this research field to open new avenues in orthodontics for using miRNAs also in a new therapeutic key.

## 5. Conclusions

miRNAs play a crucial role in the contemporary molecular biological field as current studies drive the search of new frontiers. Numerous important studies see miRNAs as protagonists in various pathologies, such as psoriasis, skin and kidney tumors, in numerous autoimmune pathologies, in metabolic-syndrome associated with diabetes and in the expression of leukotrienes in course of viral infections, which has taken a leading role in recent years with the COVID-19 pandemic [109,110,111,112,113,114]. The present review supports new studies in order to better understand and classify the various miRNAs that have already studied in other pathologies of the oral cavity, in another context and to predict the possible differences of the OTM in patients with other periodontal diseases. We made the decision to write this literature evaluation in light of the newly discovered relationships between miRNAs and the bone remodeling process. Since an orthodontic treatment has a duration greater than or equal to 2 years, the study of microRNAs involved in OTM process can provide an additional tool for clinician to personalize the orthodontic treatment (by better controlling of teeth anchorage and/or by allowing some patients the application of different degree of force), thus obtaining better results and in shorter times. 

In conclusion, further studies are needed to fully understand the role of miRNAs during OTM and, in particular, we suggest:

- To study the miRNAs involved in other orthodontic movements (e.g.: extrusion, up-righting, torque, etc.) or between orthodontic and orthopedic movements (Rapid Maxillary Expansion (RME));
- To investigate the role of miRNA34a in the improvement of anchorage or other miRNAs involved in this orthodontic system. This could improve the force used during a rapid palate expansion (RME) treatment method, as it could provide guidance on the interdigitation degree of the median palatine suture and thus reduce possible side effects on the roots of the upper first molars or prevent their exit from the alveolar bone.
- To perform further studies to evaluate the modulation of miRNAs in the very early stages following the application of a predeterminated force to understand when the bone remodeling process begins, if there is a greater risk of undergoing root resorption or after how long it takes reduce or increase strength to optimize OTM.
- Conduct clinical investigations to measure the amount of miRNAs present in the crevicular fluid on either side of the tooth. This can give a more precise evaluation of how different the miRNA under investigation is from the standard.
- Use CGF sampling to evaluate miRNAs linked to OTM as salivary samples may not be accurate enough due to the possibility of other systemic factors.

This will give a foundation for comprehending particular biological interactions that contribute to OTM and could expand the range of precision medicines and research directions in this area. Additionally, the miRNAs modulation producing iatrogenic adverse effects like caries risk or apical root resorption can be found. These measures could open new frontiers in the field of orthodontics to be able to create increasingly personalized and efficient treatment programs. In conclusion, expanding knowledge at this topic may provide new means of speeding up orthodontic treatment with minimal iatrogenic side-effects. In order to encourage the development of new research that can help us better understand the clinical roles and applications of miRNAs as markers of OTM, it is important to consider the results of our review as additional evidence of their importance in the perspective for a new view of the orthodontic field.

## Figures and Tables

**Table 1:** Identified miRNAs in OTM.

miRNA	Role in OTM	Type of Study	Biological Sample Tested	References
*miR-34a, miR-146a*	inhibition of osteoblast differentiation by downregulation of CELF3	In vitro	PDLSCs obtained from PDL tissue scraped off from the middle third of the premolar root	[38]
*miR-21*	Stimulation of OC differentiation by downregulation of PDCD4	In vivo	Rats divided in four groups: TM, PAOO, agomiR-21 and antagomir-21	[35]
*miR-34*	Promotion of OTM by targeting MMPs	Clinical trial, in vitro	CGF samples performed on the canines of patients subject to orthodontic treatment + hPDL	[32]
*miR-155-5p*	inhibition of OC differentiation by targeting CXCR2	Clinical trial	Patients with different degree of root resorption	[40]
*miR-21*	Directly promotion of OC differentiation during OTM	In vivo	WT C57BL/6 mice and miR-21^−/−^ mice	[36]
*miR-21*	Promotion of OC differentiation by promoting the RANK level secreted by T cells	In vivo	WT C57BL/6 mice and MiR-21^−/−^ mice	[37]
*miR-29*	Promotion of OC differentiation	Clinical trials	GCF sample performed during canine retraction	[31]
*miR-125a-5p*	Promotion of bone healing by targeting ETV6	In vitro	Periodontal ligament cells were isolated from normal human impacted third molars	[41]
*miR-503-5p*	Inhibition of osteogenic differentiation and bone formation in OTM tension sides	In vitro, In vivo	BMSCs and Rats (the left maxillary first molars were mesially stretched	[42]
*miR-3198*	Downregulation of OPG expression in response to mechanical stress.	In vitro	hPDL subjected to compression force (2 g/cm^2^) or tension force (15% elongation)	[43]
*miR-34a*	Promotion osteogenic differentiation by targeting GSK-3beta under orthodontic force	In vitro, in vivo	Rat bone mineral stem cells (rBMSCs) and local alveolar bone tissue. Rats (maxillary bone)	[39]
*miR-221-3p,* *miR-138-5p,* *miR-132-3p,* *miR-218-5p,* *miR-133a-3p,* *miR-145-3p,* *miR-143-5p,* *miR-486-3p,* *miR-21-3p*	Osteogenesis-related by targeting some genes (YAP1, WWTR1, TEAD2, CTGF, DVL2 and GDF5)	in vitro and in vivo	PDLCs obtained from the middle one-third (in vitro) and rats in which a coil spring connected the incisors to the first molar: after 3 days the PDL tissue from each rat’s distal stretched side and the control side was collected for RT-qPCR analysis (in vivo)	[44]

OC = Osteoclast; PDL = Periodontal Ligament; PDLSCs = Periodontal Ligament Stem Cells; OTM = Orthodontic Movements; ETV6 = E26 transformation-specific variant 6 gene; BMSCs = Bone Mesenchymal stem cells.

## Data Availability

Data are available from the corresponding author upon reasonable request.
